# Plasma Symmetric Dimethylarginine and Mortality in Kidney Transplant Recipients: A Cohort Study

**DOI:** 10.34067/KID.0000001204

**Published:** 2026-05-04

**Authors:** Daan Kremer, Dion Groothof, Sovia Salamah, Adrian Post, Tim J. Knobbe, Sarah Peterson, Marco van Londen, Murthy Yerramilli, Stefan P. Berger, Firas F. Alkaff, Stephan J.L. Bakker

**Affiliations:** 1 Division of Nephrology, Department of Internal Medicine, University Medical Center Groningen, University of Groningen, Groningen, The Netherlands; 2 Department of Public Health and Preventive Medicine, Faculty of Medicine Universitas Airlangga, Surabaya, Indonesia; 3 IDEXX Laboratories, Inc, One IDEXX Drive, Westbrook, Maine; 4 Division of Pharmacology and Therapy, Department of Anatomy, Histology, and Pharmacology, Faculty of Medicine Universitas Airlangga, Surabaya, Indonesia

**Keywords:** cardiovascular disease, chronic inflammation, kidney transplantation, mortality, oxidative stress, renal transplantation, urea, uremia, clinical science

## Abstract

**Key Points:**

- In this large cohort of kidney transplant recipients, plasma symmetric dimethylarginine concentrations were independently associated with a higher mortality risk
- Symmetric dimethylarginine may not merely be an inert uremic solute and may be involved as a uremic toxin in ongoing pathological processes

**Background:**

Long-term outcome after kidney transplantation remains limited because of high risks of cardiovascular, infectious, and malignant premature death. Uremic solutes, such as urea and symmetric dimethylarginine (SDMA), are suggested to contribute to higher oxidative stress, chronic low-grade inflammation, and excess mortality. Therefore, we evaluated the associations of urea and SDMA with all-cause and cause-specific mortality in kidney transplant recipients.

**Methods:**

Baseline plasma SDMA was measured using liquid chromatography-mass spectrometry in kidney transplant recipients enrolled in the prospective TransplantLines Food and Nutrition Biobank and Cohort Study (Groningen, The Netherlands). We assessed prospective associations of SDMA with all-cause and cause-specific mortality using Cox regression.

**Results:**

We included 628 adult kidney transplant recipients (56% male, age, 53±13 years; eGFR, 52±20 ml/min per 1.73 m^2^) at 5.4 [interquartile range, 1.8–12.0] years after transplantation. Median plasma urea was 9.6 [7.3–13.4] mmol/L, and plasma SDMA was 20 [15–25] *µ*g/dl. In Cox regression analyses, both plasma urea (hazard ratio per doubling, 2.21; 95% confidence interval, 2.22 to 2.85) and plasma SDMA (hazard ratio per doubling, 2.69; 95% confidence interval, 1.95 to 3.70) were strongly associated with a higher risk of all-cause mortality. These associations remained for SDMA but not urea in a fully adjusted model, and the observed associations were generally similar for all causes of death.

**Conclusions:**

Plasma urea and SDMA are both associated with a higher mortality risk in outpatient kidney transplant recipients. In a model including both urea and SDMA, only the association of SDMA with mortality remained.

**Clinical Trial registry name and registration number:**

NCT02811835.

## Visual Abstract

**Figure:**
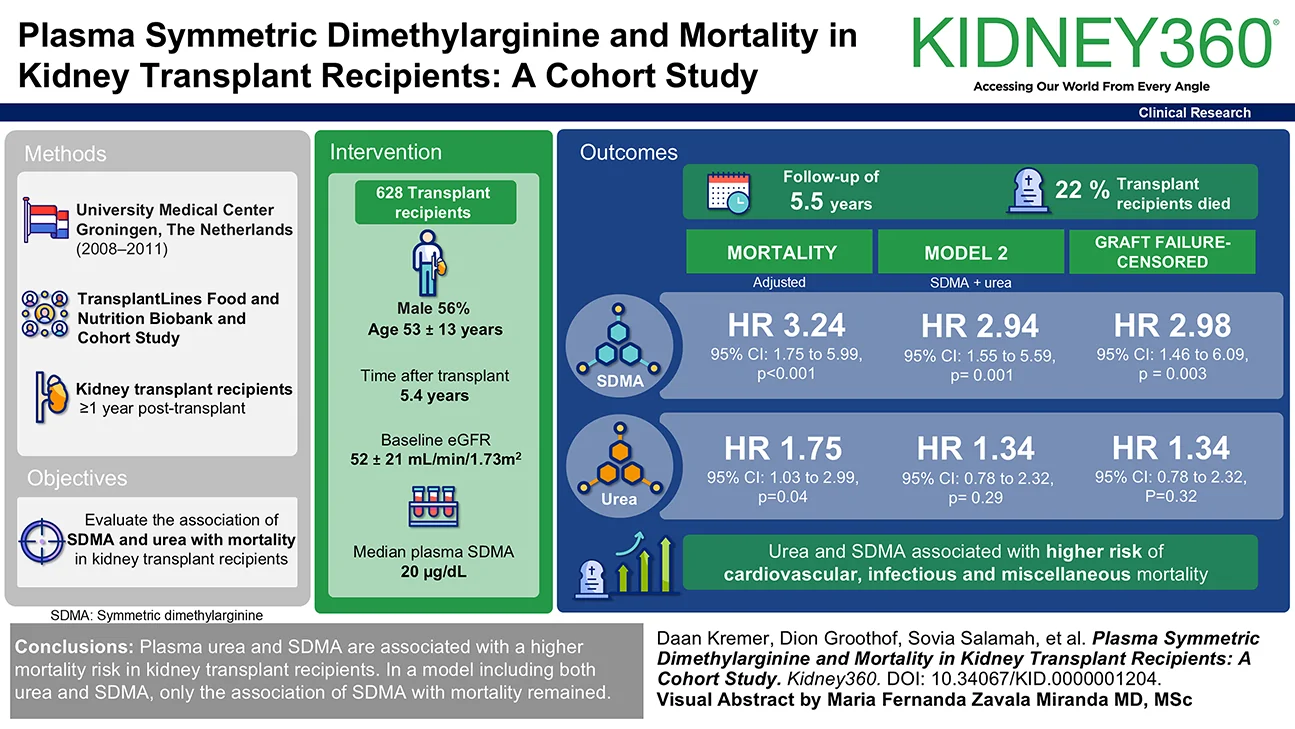

## Introduction

Kidney transplant recipients are at high risk of premature death. Some of this risk may be attributable to uremic solutes accumulating because of kidney failure.^1^ The most prominent and widely measured uremic solute is plasma urea (often referred to as BUN in the United States, where BUN=plasma urea×0.467). Urea is a small water-soluble molecule that is freely filtered by the glomerulus, followed by partial tubular reabsorption. As such, urea was recognized as a kidney function marker in the 19th century.^1^ More recently, urea was recognized as a marker of protein catabolism.^2^ While initially, urea was considered inert, accumulating evidence suggests detrimental effects of urea on insulin resistance, endothelial dysfunction, atherosclerosis, and oxidative stress.^1,3–6^

Symmetric dimethylarginine (SDMA) is another well-established uremic solute. It is an endogenous methylated arginine derivative formed during protein turnover. Unlike creatinine, the most commonly used kidney function marker in humans, SDMA is produced in almost all nucleated cells, causing its circulating concentrations to be unrelated to muscle mass.^7^ Because in veterinary medicine between-subject variation in muscle mass is an even larger problem in assessing kidney function than in human medicine, SDMA is the marker most commonly used for assessing kidney function in this field.^7^ Similar to urea, circulating SDMA concentrations depend largely on kidney function.^8^ Indeed, SDMA concentrations are elevated in patients with kidney disease and show a reduction—but do not normalize—after kidney transplantation.^8,9^ In recent decades, SDMA has been identified as a uremic toxin, showing adverse effects instead of just being an inert kidney function marker. For example, SDMA was shown to affect nitric oxide metabolism thereby exacerbating oxidative stress and causing inflammation in kidney disease through activation of NF-κB.^10,11^ Moreover, recent evidence highlighted that SDMA may be a marker of vascular dysfunction in patients with CKD.^12,13^

Considering the putative pathophysiological effects of urea and SDMA, we studied the associations of these uremic solutes with mortality in kidney transplant recipients.

## Methods

This study was reported following the Strengthening the Reporting of Observational studies in Epidemiology Guidelines (Supplemental Table 1).

### Study Population

For this study, we used data from the TransplantLines Food and Nutrition Biobank and Cohort Study, which included outpatient kidney transplant recipients with a functioning graft approximately 1 year or longer after transplantation.^14^ This cohort study was performed in the University Medical Center Groningen (Groningen, The Netherlands) between November 2008 and June 2011. The study was approved by the University Medical Center Groningen Institutional Review Board (METc 2008/186) and adheres to the Declarations of Helsinki and Istanbul (NCT02811835). All patients provided written informed consent. All participants with available plasma samples were included in the current analyses. The flow of the study population is presented in Supplemental Figure 1. The end of follow-up was defined as either patient death or reaching the censoring date of September 30th, 2015. There were no losses to follow-up.

### Data Collection and Definitions

All study measurements were performed during a morning visit to the outpatient clinic. BP was measured using a semiautomatic device following a strict protocol. Demographic data, clinical characteristics, and medical history were extracted from medical files. Diabetes was defined using the American Diabetes Association criteria. Kidney function was assessed using the creatinine-based eGFR, according to the 2009 CKD-Epidemiology Collaboration equation.

### Baseline and End Points

In this study, baseline (time 0) was defined as the moment of blood sampling, that is at least 1 year after transplantation.

The end point of this study was mortality. There were no losses to follow-up.

### Biochemical Analyses

Blood was drawn at baseline after an overnight fasting period of at least 8 hours, including no medication use, and then stored at −80°C until analysis. SDMA concentrations were measured in EDTA-plasma using liquid chromatography-mass spectrometry by IDEXX Laboratories, Inc (Westbrook, ME). Other clinical chemistry assays including plasma urea were performed using automated routine laboratory methods (Roche Diagnostics, Basel, Switzerland).

### Statistical Analyses

Baseline characteristics were summarized. Linear regression was used to assess the associations of plasma SDMA and urea with other parameters, where parameters were log_2_ transformed if necessary to meet assumptions of linearity and homoscedasticity (in linear regression analyses). Regression coefficients were given as standardized beta (St. *β*) values, referring to the number of SDs a dependent variable changes per one SD increase of the independent variable. This allows for a better comparison of the strengths and the potential effect sizes of the different potential determinants. Cox proportional hazards regression analyses were applied to investigate the associations of plasma SDMA and urea with mortality. In multivariable Cox regression analyses, we included variables known to be associated with mortality and/or uremia in this population, including age, sex, eGFR, diabetes, preemptive transplantation, time after transplantation, donor status (living versus deceased), donor age, history of cardiovascular disease, rejection, 24-hour urinary protein excretion, use of calcineurin inhibitors and diuretics, hemoglobin, albumin, high-sensitivity *C*-reactive protein, *N*-terminal brain natriuretic peptide (NT-proBNP), and urea (in case of SDMA) or SDMA (in case of urea). Potential effect modification by age, sex, eGFR, and 24-hour urinary protein excretion on the prospective associations of plasma SDMA were assessed by adding interaction terms to the final models but were not observed. In Cox regression analyses, hazard ratios (HR) are presented per log_2_ increment, allowing for easy interpretation, as corresponding HRs are interpretable as coefficients per doubling of the variable of interest.

In sensitivity analyses, we assessed the associations of urea and SDMA with graft failure-censored mortality as the dependent variable in Cox regression analyses. Moreover, in exploratory analyses, we repeated the Cox regression analyses with death due to cardiovascular disease, infection, malignancy, or miscellaneous causes.

Schoenfeld residuals were visually checked, and the final models did not violate the assumption for proportionality of hazards. HRs are presented per doubling of SDMA and urea, with 95% confidence intervals (95% CI). The data were analyzed using IBM SPSS 23.0 (SPSS Inc., Chicago, IL) and *R* version 4.0.5 (Vienna, Austria). For all analyses, *P* < 0.05 was considered to indicate statistical significance.

## Results

### Baseline Characteristics

Baseline characteristics according to tertiles of SDMA are presented in Supplemental Table 2 and in the overall group in Table 1. We included 628 kidney transplant recipients (56% male, age 53±13 years) at a median [interquartile range] time after transplantation of 5.2 [1.8–12.0] years. Baseline eGFR was 52±21 ml/min per 1.73 m^2^, and plasma SDMA concentration was 20 [15–25] *µ*g/dl.

**Table 1:** Baseline characteristics and linear regression analyses of the 628 included kidney transplant recipients with log_2_ symmetric dimethylarginine or log_2_ urea as the dependent variable

Variable	*N*=628	St. *β* with log_2_ SDMA	St. *β* with log_2_ Urea
Plasma SDMA (*µ*g/dl)	20 [15–25]	N/A	0.77 (0.72 to 0.82)
Urea (mmol/L)	9.6 [7.3–13.4]	0.77 (0.72 to 0.82)	N/A
BUN (mg/dl)	26.9 [20.4–37.5]	0.77 (0.72 to 0.82)	
**Clinical parameters**			
Female sex (*n*, %)	274 (44)	−0.23 (−0.38 to −0.07)	−0.08 (−0.23 to 0.07)
Age at visit (yr)	53 (13)	−0.03 (−0.11 to 0.05)	0.09 (0.02 to 0.17)
Preemptive transplantation (*n*, %)	99 (16)	−0.22 (−0.43 to −0.00)	−0.24 (−0.45 to −0.04)
Time after transplantation (yr)^a^	5.3 [1.8–11.9]	0.03 (−0.05 to 0.11)	−0.03 (−0.10 to 0.05)
History of rejection (*n*, %)	169 (27)	0.38 (0.20 to 0.55)	0.35 (0.18 to 0.51)
Primary renal disease (*n*, %)			
*GN*	164 (26)	0.11 (−0.14 to 0.36)	0.14 (−0.09 to 0.38)
*Interstitial nephritis*	83 (13)	−0.06 (−0.35 to 0.23)	0.18 (−0.07 to 0.43)
*Cystic kidney disease*	127 (20)	0.17 (−0.09 to 0.43)	0.18 (−0.07 to 0.43)
*Other congenital/hereditary disease*	31 (5)	−0.02 (−0.42 to 0.38)	−0.09 (−0.46 to 0.27)
*Renal vascular disease*	33 (5)	0.17 (−0.22 to 0.56)	0.20 (−0.15 to 0.57)
*Diabetes*	29 (5)	0.59 (0.18 to 1.00)	0.68 (0.31 to 1.05)
*Other multisystem diseases*	44 (7)	0.03 (−0.33 to 0.38)	0.04 (−0.30 to 0.38)
*Other or unknown cause*	117 (19)	−0.04 (−0.55 to 0.48)	−0.16 (−0.64 to 0.33)
Diabetes (*n*, %)	151 (24)	−0.01 (−0.19 to 0.17)	0.16 (−0.01 to 0.33)
History of CV disease (*n*, %)	149 (24)	0.06 (−0.13 to 0.24)	0.15 (−0.02 to 0.32)
Systolic BP (mm Hg)	136 (17)	0.09 (0.01 to 0.17)	0.12 (0.05 to 0.20)
Current smoking (*n*, %)	73 (12)	0.09 (−0.16 to 0.33)	0.07 (−0.16 to 0.30)
Donor age (yr)	43 (15)	0.19 (0.11 to 0.27)	0.19 (0.11 to 0.26)
Living donor (*n*, %)	221 (35)	−0.28 (−0.44 to −0.11)	−0.21 (−0.04 to −0.06)
**Laboratory measurements**			
Leukocytes (×10^9^/L)	8.2 (2.7)	−0.02 (−0.10 to 0.06)	−0.02 (−0.09 to 0.06)
Hemoglobin (g/dl)	13.2 (1.81)	−0.38 (−0.46 to −0.31)	−0.42 (−0.49 to −0.35)
Sodium (mmol/L)	141 (3)	−0.17 (−0.24 to −0.09)	−0.11 (−0.18 to −0.03)
Potassium (mmol/L)	4.0 (0.5)	0.35 (0.27 to 0.42)	0.36 (0.29 to 0.42)
eGFR (ml/min per 1.73 m^2^)	52 (21)	−0.79 (−0.84 to −0.74)	−0.80 (−0.85 to −0.76)
Creatinine (mg/dl)^a^	1.40 [1.12–1.81]	0.82 (0.78 to 0.87)	0.79 (0.75 to 0.84)
hs-CRP (mg/L)^a^	1.6 [0.7–4.4]	0.06 (−0.02 to 0.14)	0.04 (−0.04 to 0.11)
24-h urinary protein excretion (g/24 h)^a^	0.2 [0.0–0.4]	0.22 (0.14 to 0.30)	0.23 (0.15 to 0.30)
**Medication use (*n*, %)**			
Diuretics (*n*, %)	251 (40)	0.55 (0.39 to 0.70)	0.67 (0.53 to 0.82)
RAAS-blocker (*n*, %)	310 (49)	0.25 (0.10 to 0.41)	0.36 (0.21 to 0.50)
Calcium-antagonist (*n*, %)	154 (25)	0.06 (−0.12 to 0.24)	0.11 (−0.07 to 0.28)
*β* blocker (*n*, %)	398 (63)	0.15 (−0.01 to 0.31)	0.27 (0.12 to 0.42)
Prednisolone (*n*, %)	621 (99)	−0.28 (−1.03 to 0.47)	−0.58 (−1.33 to 0.16)
Calcineurin inhibitor (*n*, %)	371 (59)	0.51 (0.35 to 0.66)	0.56 (0.42 to 0.71)
Proliferation inhibitor (*n*, %)	521 (83)	−0.35 (−0.56 to −0.14)	0.30 (−0.49 to −0.10)
mTOR inhibitor (*n*, %)	20 (3)	−0.45 (−0.89 to 0.00)	−0.42 (−0.82 to −0.01)

Data on donor age, smoking status, and hs-CRP were missing in 17 patients (3%), 42 patients (7%), and 37 patients (6%), respectively. All other variables were missing in less than three patients. Normally distributed data are presented as mean±SD, skewed data as median [interquartile range], and categorical data as number (valid percentage). CV, cardiovascular; eGFR, eGFR as calculated using CKD-Epidemiology Collaboration formula; hs-CRP, high-sensitivity *C*-reactive protein; mTOR, mechanistic target of rapamycin; RAAS, renin-angiotensin-aldosterone system; SDMA, symmetric dimethyl arginine.

a Variables were log_2_ transformed.

### Linear Regression Analyses

Linear regression analyses are presented in Table 1. In brief, higher creatinine (St. *β*, 0.79; 95% CI, 0.75 to 0.84), higher SDMA (St. *β*, 0.77; 95% CI, 0.72 to 0.82), use of diuretics (St. *β*, 0.67; 95% CI, 0.53 to 0.82), calcineurin inhibitors (St. *β*, 0.56; 95% CI, 0.42 to 0.71), and history of rejection (St. *β*, 0.35; 95% CI, 0.18 to 0.51) were among the variables that were most strongly associated with higher plasma urea. Similar to urea, higher creatinine (St. *β*, 0.82; 95% CI, 0.78 to 0.87), higher urea (St. *β*, 0.77; 95% CI, 0.72 to 0.82), use of diuretics (St. *β*, 0.55; 95% CI, 0.39 to 0.70), calcineurin inhibitors (St. *β*, 0.51; 95% CI, 0.35 to 0.66), and history of rejection (St. *β*, 0.38; 95% CI, 0.20 to 0.55) were associated with higher plasma SDMA.

### Survival Analyses

During a median follow-up of 5.5 [4.7–6.1] years, 138 kidney transplant recipients (22%) died. Kaplan–Meier curves visualizing differences in survival across SDMA and urea tertiles are presented in Supplemental Figure 2.

In Cox regression analyses, plasma urea was associated with mortality (HR per doubling, 2.21; 95% CI, 2.22 to 2.85; Table 2). This association remained significant after adjustment for age, sex, time after transplantation, eGFR, donor status, donor age, preemptive transplantation, rejection, 24-hour urinary protein excretion, diabetes, history of cardiovascular disease, use of diuretics and calcineurin inhibitors, albumin, hemoglobin, *C*-reactive protein, and NT-proBNP (HR per doubling, 1.75; 95% CI, 1.03 to 2.99).

**Table 2:** Cox regression analysis of the association of plasma symmetric dimethylarginine and urea with mortality

	Urea		SDMA	
Model	HR per Doubling (95% CI)	*P* Value	HR per Doubling (95% CI)	*P* Value
Crude	2.21 (1.71 to 2.85)	<0.001	2.69 (1.95 to 3.70)	<0.001
Model 1	2.19 (1.68 to 2.86)	<0.001	3.06 (2.19 to 4.28)	<0.001
Model 2	2.41 (1.50 to 3.87)	<0.001	4.67 (2.53 to 8.59)	<0.001
Model 3	2.19 (1.34 to 3.58)	<0.001	4.22 (2.31 to 7.69)	<0.001
Model 4	2.11 (1.28 to 3.50)	0.004	4.00 (2.20 to 7.28)	<0.001
Model 5	2.11 (1.26 to 3.51)	0.005	4.00 (2.19 to 7.32)	<0.001
Model 6	1.75 (1.03 to 2.99)	0.04	3.24 (1.75 to 5.99)	<0.001
Model 7	1.34 (0.78 to 2.32)	0.29	2.94 (1.55 to 5.59)	0.001

During follow-up, 138 patients (22%) experienced death during the median follow-up of 5.5 [4.7 to 6.1] years. Model 1, adjustment for age, sex and time after transplantation. Model 2, adjustment for variable in model 1+creatinine-based eGFR+living versus deceased donor status+donor age+preemptive transplantation. Model 3, adjustment for model 2+rejection+24-hour urinary protein excretion. Model 4, adjustment for model 3+diabetes+history of cardiovascular disease. Model 5, adjustment for model 4+diuretic use+calcineurin inhibitor use. Model 6, adjustment for model 5+albumin+hemoglobin+high-sensitivity *C*-reactive protein+*N*-terminal probrain natriuretic peptide. Model 7, adjustment for model 6+urea (in case of symmetric dimethylarginine) or symmetric dimethylarginine (in case of urea). CI, confidence interval; HR, hazard ratio; SDMA, symmetric dimethyl arginine.

In similar analyses, plasma SDMA was also associated with mortality (HR per doubling, 2.69; 95% CI, 1.95 to 3.70), which strengthened after adjustment for the aforementioned potential confounders (HR per doubling, 3.24; 95% CI, 1.75 to 5.99).

**Figure 1:**
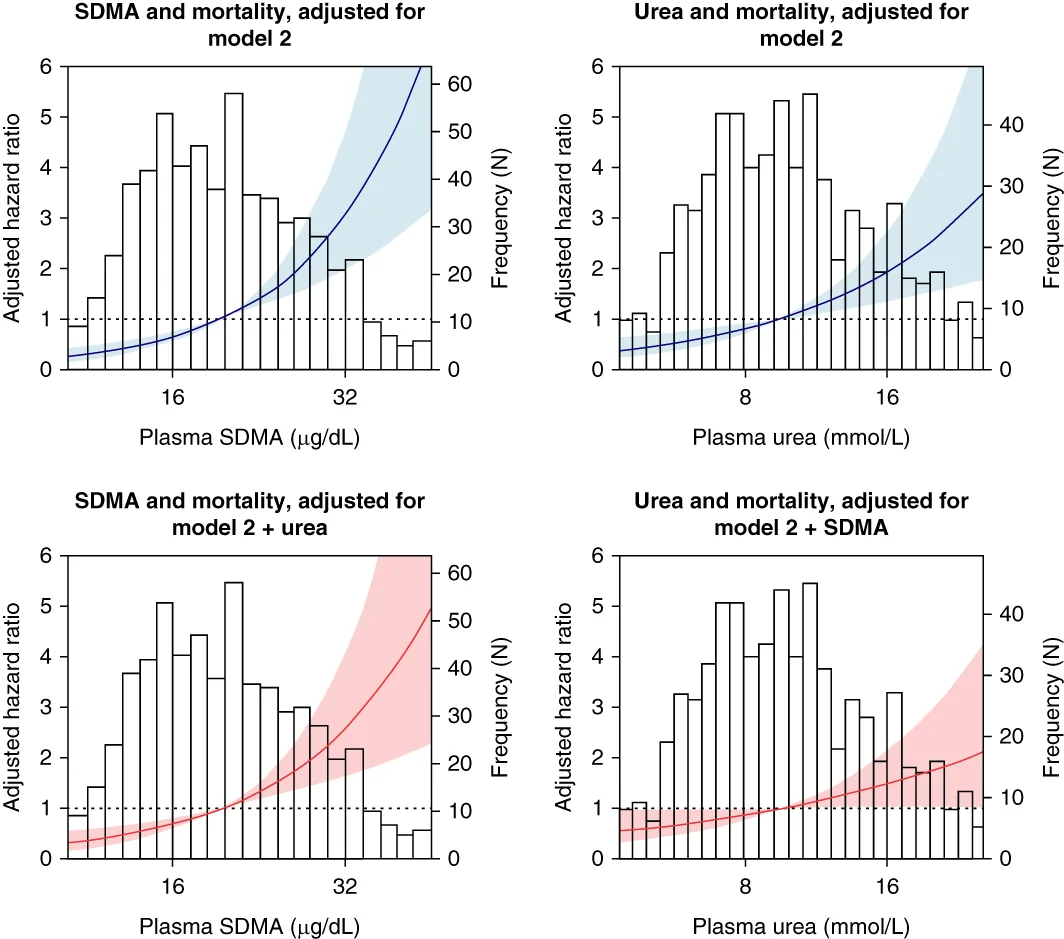
**Association of SDMA (left) and urea (right) with mortality after Cox regression adjusted for model 2, including age, sex, time after transplantation, eGFR, preemptive transplantation, donor type, donor age, and preemptive transplantation, and in a model including adjustments for model 2+urea/SDMA (bottom figures).** SDMA, symmetric dimethyl arginine.

Interestingly, as visualized in Figure 1, plasma SDMA (HR per doubling, 2.94; 95% CI, 1.55 to 5.59; *P* = 0.001) but not urea (HR per doubling, 1.34; 95% CI, 0.78 to 2.32; *P* = 0.29) was significantly associated with mortality in a model encompassing both uremic solutes.

### Sensitivity Analyses with Graft Failure-censored Mortality

In total 27 kidney transplant recipients died after development of graft failure. To excluded the possibility that death after development of graft failure is a main driver underlying the associations that we found, we performed sensitivity analyses with mortality censored for graft failure. In total, 111 patients (18%) experience death when censored for graft failure during a median follow-up of 5.4 [4.5–6.0] years. Similar to the primary analyses, both urea and SDMA were associated with higher risks of graft failure-censored mortality, as shown in Supplemental Table 3. Again, similar to the primary analyses, SDMA (HR per doubling, 2.98; 95% CI, 1.46 to 6.09, *P* = 0.003) but not urea (HR per doubling, 1.34; 95% CI, 0.78 to 2.32, *P* = 0.32) was significantly associated with mortality in a model encompassing both uremic solutes.

### Explorative Analyses with Cause-specific Mortality

In additional explorative analyses we assessed the associations of urea and SDMA with cause-specific mortality (Supplemental Table 4). In total, 51 patients (8%) experienced death from cardiovascular causes, 41 (6%) from infectious causes, 23 (4%) from malignancy, and 23 (4%) from miscellaneous causes during the median follow-up of 5.5 [4.7–6.1] years. Despite numerical changes and limited power, both urea and SDMA were associated with a higher risk of cardiovascular, infectious, and miscellaneous mortality (*P* ≤ 0.01 for all, respectively). Although statistically insignificant, there were trends toward associations of higher SDMA and urea with risk of mortality due to malignancy (*P* > 0.05 for both).

Upon adjustment for age and sex, the associations remained materially unchanged. In a model encompassing age, sex, and both urea and SDMA, the direction of the observed associations remained, although the statistical significance of the associations remained only for the association of urea with cardiovascular mortality, and of SDMA with infectious mortality.

## Discussion

In this large cohort of outpatient kidney transplant recipients, plasma urea and SDMA concentrations were associated with a higher mortality risk independent of multiple confounders. However, the association of plasma urea with mortality lost significance, whereas the association of plasma SDMA with mortality remained strong in a model including both uremic toxins.

Initially, it may seem that the links between urea, SDMA, and mortality stem from residual kidney function. However, the estimated HRs of SDMA with mortality were higher upon adjustment for kidney function parameters, suggesting that the associations of SDMA with outcomes reflect not merely kidney function but also other pathological processes. The notion that uremic toxins may reflect other detrimental pathological processes is supported by preclinical and clinical evidence, including suggested roles in insulin resistance, endothelial dysfunction, and atherosclerosis and oxidative stress.^10,15^ Indeed, the suggested pathological effects of high SDMA concentrations are also reflected in epidemiological studies, which showed consistent associations of SDMA with cardiovascular disease and death.^16^

Moreover, associations between SDMA and mortality after adjustment for creatinine-based parameters of kidney function were also observed in sepsis and coronavirus disease 2019.^17,18^ Similarly, SDMA was also associated with mortality from cardiovascular causes in other populations.^16^ These observations may be explained by experimental evidence, which has suggested that SDMA can impair nitric oxide synthesis, stimulate the generation of reactive oxygen species, and contribute to proinflammatory events in the vascular wall and monocyte activation.^15^ All in all, these findings support the notion that SDMA may not only be an innocent bystander and reflection of impaired kidney function but may also exert detrimental effects on patient outcomes.^11^ These findings stress the relevance of specific uremic toxins such as SDMA, rather than uremia itself.

Exploratory analyses with cause-specific mortality showed that the trends of the association of urea and SDMA with mortality was similar to the primary analyses for all causes, although statistical significance varied depending on statistical power. Interestingly, the trends of SDMA regarding the associations with death due to malignancy, infectious and miscellaneous causes of death were much more apparent compared with urea. Although the limited power for these analyses prohibits any definite conclusions, this observation suggests that SDMA is involved in detrimental processes beyond kidney function and cardiovascular risk alone. This suggestion was corroborated in sensitivity analyses with graft failure–censored mortality, rather than uncensored mortality. In these analyses, the results remained materially unchanged, suggesting that the observed associations are not merely a reflection of kidney function in these patients.

A strength of the current study is the extensive phenotypical data available, allowing for adjustment for many potential confounders, including 24-hour urinary protein excretion, medication use, history of cardiovascular disease, and NT-proBNP. The observation that the associations between SDMA remained independent of these potential confounders suggests that SDMA may be implicated in detrimental processes beyond kidney function and/or volume status. Moreover, the sensitivity analyses confirmed that the associations with mortality are independent of graft failure.

It should be realized, however, that because of the observational study design, we cannot provide causal evidence for any potential detrimental effects of SDMA in the body that may explain the observed associations with mortality. In addition, our study included a predominantly White Dutch population, which necessitates validation of our study results in other populations. Although SDMA is considered less muscle-dependent than, for example, creatinine, its applicability may be less hampered when extrapolating to non-European populations. However, future research in different populations is needed to confirm this. Moreover, future studies are needed to assess the potential detrimental effects of other uremic toxins. In addition, the lack of serial SDMA measurements and the possibility of residual unmeasured confounding (*e.g*., donor-specific antibodies) inherent to the observational nature of the study limits its interpretation. Moreover, future studies may adjust for other assessments of kidney function, such as iothalamate-measured GFR or cystatin *C*-based glomerular filtration estimates, especially considering recent publications highlighting that the difference in cystatin *C* versus creatinine-based estimated glomerular filtration also yields prognostic information for mortality.^19^

In conclusion, in this large cohort of kidney transplant recipients, plasma SDMA concentrations were associated with a higher mortality risk. Interestingly, these associations strengthened upon adjustment for kidney function, suggesting that SDMA is not an inert uremic retention solute and may be involved in additional ongoing pathological processes.

## Supplementary Material

## Data Availability

Original data cannot be shared. Detailed Explanation Why Data Cannot Be Shared: Data sharing was not included in the study's informed consent.

## Declarative Statements

This study includes clinical experimentation and received Institutional Review Board or Ethics Committee approval. All patients provided written informed consent. This study includes clinical experimentation and complies with the Declaration of Helsinki. The clinical and research activities being reported are consistent with the Principles of the Declaration of Istanbul as outlined in the Declaration of Istanbul on Organ Trafficking and Transplant Tourism.

## Supplemental Material

Supplemental Table 1. STROBE statement—checklist of items that should be included in reports of observational studies.

Supplemental Table 2. Baseline characteristics of 628 kidney transplant recipients in tertiles of plasma SDMA.

Supplemental Table 3. Cox regression analysis of the association of plasma SDMA and urea with graft failure-censored mortality.

Supplemental Table 4. Cox regression analysis of the association of plasma SDMA with cause-specific mortality.

Supplemental Figure 1. Diagram visualizing the flow of participants through the study.

Supplemental Figure 2. Kaplan–Meier curve visualizing differences in survival across SDMA and urea tertiles.
