# Supplementary material for: Plasma Symmetric Dimethylarginine and Mortality in Kidney Transplant Recipients: A Cohort Study
Source: Kidney360. 2026 May 4;7(8):1873–9. doi: 10.34067/KID.0000001204 (PMC13567852; doi:10.34067/KID.0000001204)
Supplement: Supplementary file 1 [file kidney360-7-1873-s001.pdf]

ASN American Society of Nephrology | JASN Journal of the American Society of Nephrology | KIDNEY360 Accessing Our World From Every Angle | CJASN Clinical Journal of the American Society of Nephrology

ASN Journal Disclosure Form

As per ASN journal policy, I have disclosed any financial relationships or commitments I have held in the past 36 months as included below. I have listed my Current Employer below to indicate there is a relationship requiring disclosure. If no relationship exists, my Current Employer is not listed.

F. Alkaff reports the following:
Employer: University Medical Center Groningen

I understand that the information above will be published within the journal article, if accepted, and that failure to comply and/or to accurately and completely report the potential financial conflicts of interest could lead to the following: 1) Prior to publication, article rejection, or 2) Post-publication, sanctions ranging from, but not limited to, issuing a correction, reporting the inaccurate information to the authors' institution, banning authors from submitting work to ASN journals for varying lengths of time, and/or retraction of the published work.

Name: Firas F. Alkaff
Manuscript ID: K360-2025-001428R1
Manuscript Title: Plasma Symmetric Dimethylarginine and Mortality in Kidney Transplant Recipients: A Cohort Study
Date of Completion: February 13, 2026
Disclosure Updated Date: February 13, 2026

ASN American Society of Nephrology
JASN Journal of the American Society of Nephrology
KIDNEY360 Accessing Our World From Every Angle
CJASN Clinical Journal of the American Society of Nephrology

ASN Journal Disclosure Form

As per ASN journal policy, I have disclosed any financial relationships or commitments I have held in the past 36 months as included below. I have listed my Current Employer below to indicate there is a relationship requiring disclosure. If no relationship exists, my Current Employer is not listed.

S. Bakker reports the following:
Employer: University Medical Center Groningen; and Research Funding: Astellas Pharma, Chiesi, bioMerieux.

I understand that the information above will be published within the journal article, if accepted, and that failure to comply and/or to accurately and completely report the potential financial conflicts of interest could lead to the following: 1) Prior to publication, article rejection, or 2) Post-publication, sanctions ranging from, but not limited to, issuing a correction, reporting the inaccurate information to the authors' institution, banning authors from submitting work to ASN journals for varying lengths of time, and/or retraction of the published work.

Name: Stephan J.L. Bakker
Manuscript ID: K360-2025-001428R3
Manuscript Title: Plasma Symmetric Dimethylarginine and Mortality in Kidney Transplant Recipients: A Cohort Study
Date of Completion: April 4, 2026
Disclosure Updated Date: May 9, 2025

ASN Journal Disclosure Form

As per ASN journal policy, I have disclosed any financial relationships or commitments I have held in the past 36 months as included below. I have listed my Current Employer below to indicate there is a relationship requiring disclosure. If no relationship exists, my Current Employer is not listed.

S. Berger reports the following:
Employer: UMCG; and Advisory or Leadership Role: Supervisory Board Dutch Transplant Foundation.

I understand that the information above will be published within the journal article, if accepted, and that failure to comply and/or to accurately and completely report the potential financial conflicts of interest could lead to the following: 1) Prior to publication, article rejection, or 2) Post-publication, sanctions ranging from, but not limited to, issuing a correction, reporting the inaccurate information to the authors' institution, banning authors from submitting work to ASN journals for varying lengths of time, and/or retraction of the published work.

Name: Stefan P. Berger
Manuscript ID: K360-2025-001428R1
Manuscript Title: Plasma Symmetric Dimethylarginine and Mortality in Kidney Transplant Recipients: A Cohort Study
Date of Completion: March 16, 2026
Disclosure Updated Date: March 16, 2026

ASN American Society of Nephrology | JASN Journal of the American Society of Nephrology | KIDNEY360 Accessing Our World From Every Angle | CJASN Clinical Journal of the American Society of Nephrology

ASN Journal Disclosure Form

As per ASN journal policy, I have disclosed any financial relationships or commitments I have held in the past 36 months as included below. I have listed my Current Employer below to indicate there is a relationship requiring disclosure. If no relationship exists, my Current Employer is not listed.

D. Groothof reports the following:
Employer: University Medical Center Groningen

I understand that the information above will be published within the journal article, if accepted, and that failure to comply and/or to accurately and completely report the potential financial conflicts of interest could lead to the following: 1) Prior to publication, article rejection, or 2) Post-publication, sanctions ranging from, but not limited to, issuing a correction, reporting the inaccurate information to the authors' institution, banning authors from submitting work to ASN journals for varying lengths of time, and/or retraction of the published work.

Name: Dion Groothof
Manuscript ID: K360-2025-001428R2
Manuscript Title: Plasma Symmetric Dimethylarginine and Mortality in Kidney Transplant Recipients: A Cohort Study.
Date of Completion: March 26, 2026
Disclosure Updated Date: March 26, 2026

ASN Journal Disclosure Form

As per ASN journal policy, I have disclosed any financial relationships or commitments I have held in the past 36 months as included below. I have listed my Current Employer below to indicate there is a relationship requiring disclosure. If no relationship exists, my Current Employer is not listed.

T. Knobbe reports the following:
Employer: UMCG

I understand that the information above will be published within the journal article, if accepted, and that failure to comply and/or to accurately and completely report the potential financial conflicts of interest could lead to the following: 1) Prior to publication, article rejection, or 2) Post-publication, sanctions ranging from, but not limited to, issuing a correction, reporting the inaccurate information to the authors' institution, banning authors from submitting work to ASN journals for varying lengths of time, and/or retraction of the published work.

Name: Tim J. Knobbe
Manuscript ID: K360-2025-001428R1
Manuscript Title: Plasma Symmetric Dimethylarginine and Mortality in Kidney Transplant Recipients: A Cohort Study
Date of Completion: February 13, 2026
Disclosure Updated Date: February 13, 2026

ASN Journal Disclosure Form

As per ASN journal policy, I have disclosed any financial relationships or commitments I have held in the past 36 months as included below. I have listed my Current Employer below to indicate there is a relationship requiring disclosure. If no relationship exists, my Current Employer is not listed.

D. Kremer reports the following:
Employer: University of Groningen and University Medical Centre Groningen, Groningen, The Netherlands; and Research Funding: The TransplantLines Food and Nutrition Biobank and Cohort Study (ClinicalTrials.gov; #NCT02811835) was financially supported by Top Institute Food and Nutrition (grant A-1003). In addition, this collaboration project is co-financed by the Dutch Ministry of Economic Affairs and Climate Policy by means of the PPP-allowance made available by the Top Sector Life Sciences & Health to stimulate public-private partnerships.

I understand that the information above will be published within the journal article, if accepted, and that failure to comply and/or to accurately and completely report the potential financial conflicts of interest could lead to the following: 1) Prior to publication, article rejection, or 2) Post-publication, sanctions ranging from, but not limited to, issuing a correction, reporting the inaccurate information to the authors' institution, banning authors from submitting work to ASN journals for varying lengths of time, and/or retraction of the published work.

Name: Daan Kremer
Manuscript ID: K360-2025-001428R1
Manuscript Title: Plasma Symmetric Dimethylarginine and Mortality in Kidney Transplant Recipients: A Cohort Study
Date of Completion: February 23, 2026
Disclosure Updated Date: February 23, 2026

ASN
American Society of Nephrology
JASN
Journal of the American Society of Nephrology
KIDNEY360
Accessing Our World From Every Angle
CJASN
Clinical Journal of the American Society of Nephrology

ASN Journal Disclosure Form

As per ASN journal policy, I have disclosed any financial relationships or commitments I have held in the past 36 months as included below. I have listed my Current Employer below to indicate there is a relationship requiring disclosure. If no relationship exists, my Current Employer is not listed.

S. Peterson reports the following:
Employer: IDEXX Laboratories, Inc.; Ownership Interest: IDEXX Laboratories, Inc.; and Patents or Royalties: IDEXX Laboratories, Inc.

I understand that the information above will be published within the journal article, if accepted, and that failure to comply and/or to accurately and completely report the potential financial conflicts of interest could lead to the following: 1) Prior to publication, article rejection, or 2) Post-publication, sanctions ranging from, but not limited to, issuing a correction, reporting the inaccurate information to the authors' institution, banning authors from submitting work to ASN journals for varying lengths of time, and/or retraction of the published work.

Name: Sarah M. Peterson
Manuscript ID: K360-2025-001428R1
Manuscript Title: Plasma Symmetric Dimethylarginine and Mortality in Kidney Transplant Recipients: A Cohort Study
Date of Completion: February 12, 2026
Disclosure Updated Date: February 12, 2026

ASN American Society of Nephrology | JASN Journal of the American Society of Nephrology | KIDNEY360 Accessing Our World From Every Angle | CJASN Clinical Journal of the American Society of Nephrology

ASN Journal Disclosure Form

As per ASN journal policy, I have disclosed any financial relationships or commitments I have held in the past 36 months as included below. I have listed my Current Employer below to indicate there is a relationship requiring disclosure. If no relationship exists, my Current Employer is not listed.

A. Post has nothing to disclose.

I understand that the information above will be published within the journal article, if accepted, and that failure to comply and/or to accurately and completely report the potential financial conflicts of interest could lead to the following: 1) Prior to publication, article rejection, or 2) Post-publication, sanctions ranging from, but not limited to, issuing a correction, reporting the inaccurate information to the authors' institution, banning authors from submitting work to ASN journals for varying lengths of time, and/or retraction of the published work.

Name: Adrian Post
Manuscript ID: K360-2025-001428R1
Manuscript Title: Plasma Symmetric Dimethylarginine and Mortality in Kidney Transplant Recipients: A Cohort Study
Date of Completion: February 13, 2026
Disclosure Updated Date: February 13, 2026

ASN
American Society of Nephrology
JASN
Journal of the American Society of Nephrology
KIDNEY360
Accessing Our World From Every Angle
CJASN
Clinical Journal of the American Society of Nephrology

ASN Journal Disclosure Form

As per ASN journal policy, I have disclosed any financial relationships or commitments I have held in the past 36 months as included below. I have listed my Current Employer below to indicate there is a relationship requiring disclosure. If no relationship exists, my Current Employer is not listed.

S. Salamah has nothing to disclose.

I understand that the information above will be published within the journal article, if accepted, and that failure to comply and/or to accurately and completely report the potential financial conflicts of interest could lead to the following: 1) Prior to publication, article rejection, or 2) Post-publication, sanctions ranging from, but not limited to, issuing a correction, reporting the inaccurate information to the authors' institution, banning authors from submitting work to ASN journals for varying lengths of time, and/or retraction of the published work.

Name: Sovia Salamah
Manuscript ID: K360-2025-001428R1
Manuscript Title: Plasma Symmetric Dimethylarginine and Mortality in Kidney Transplant Recipients: A Cohort Study
Date of Completion: February 14, 2026
Disclosure Updated Date: February 14, 2026

ASN American Society of Nephrology
JASN Journal of the American Society of Nephrology
KIDNEY360 Accessing Our World From Every Angle
CJASN Clinical Journal of the American Society of Nephrology

ASN Journal Disclosure Form

As per ASN journal policy, I have disclosed any financial relationships or commitments I have held in the past 36 months as included below. I have listed my Current Employer below to indicate there is a relationship requiring disclosure. If no relationship exists, my Current Employer is not listed.

M. van Londen reports the following:
Employer: University Medical Center Groningen; Frisius Medical Center Leeuwarden

I understand that the information above will be published within the journal article, if accepted, and that failure to comply and/or to accurately and completely report the potential financial conflicts of interest could lead to the following: 1) Prior to publication, article rejection, or 2) Post-publication, sanctions ranging from, but not limited to, issuing a correction, reporting the inaccurate information to the authors' institution, banning authors from submitting work to ASN journals for varying lengths of time, and/or retraction of the published work.

Name: Marco van Londen
Manuscript ID: K360-2025-001428R1
Manuscript Title: Plasma Symmetric Dimethylarginine and Mortality in Kidney Transplant Recipients: A Cohort Study
Date of Completion: February 12, 2026
Disclosure Updated Date: February 12, 2026

ASN
American Society of Nephrology
JASN
Journal of the American Society of Nephrology
KIDNEY360
Accessing Our World From Every Angle
CJASN
Clinical Journal of the American Society of Nephrology

ASN Journal Disclosure Form

As per ASN journal policy, I have disclosed any financial relationships or commitments I have held in the past 36 months as included below. I have listed my Current Employer below to indicate there is a relationship requiring disclosure. If no relationship exists, my Current Employer is not listed.

M. Yerramilli reports the following:
Employer: IDEXX Laboratories Inc; Ownership Interest: IDEXX Laboratories Inc; Research Funding: IDEXX Laboratories Inc; Patents or Royalties: IDEXX Laboratories Inc; and Advisory or Leadership Role: Vice President, R&D.

I understand that the information above will be published within the journal article, if accepted, and that failure to comply and/or to accurately and completely report the potential financial conflicts of interest could lead to the following: 1) Prior to publication, article rejection, or 2) Post-publication, sanctions ranging from, but not limited to, issuing a correction, reporting the inaccurate information to the authors' institution, banning authors from submitting work to ASN journals for varying lengths of time, and/or retraction of the published work.

Name: Murthy V. Yerramilli
Manuscript ID: K360-2025-001428R2
Manuscript Title: Plasma Symmetric Dimethylarginine and Mortality in Kidney Transplant Recipients: A Cohort Study
Date of Completion: March 20, 2026
Disclosure Updated Date: February 12, 2026
