# Supplementary material for: Plasma Symmetric Dimethylarginine and Mortality in Kidney Transplant Recipients: A Cohort Study
Source: Kidney360. 2026 May 4;7(8):1873–9. doi: 10.34067/KID.0000001204 (PMC13567852; doi:10.34067/KID.0000001204)
Supplement: Supplementary file 2 [file kidney360-7-1873-s002.pdf]

Supplemental Table 1 | STROBE Statement—checklist of items that should be included in reports of observational studies

	Item No.	Recommendation	Page No.	Relevant text from manuscript
**Title and abstract**	1	(*a*) Indicate the study's design with a commonly used term in the title or the abstract	Title + abstract	Cohort study
		(*b*) Provide in the abstract an informative and balanced summary of what was done and found	Abstract	
**Introduction**				
Background/rationale	2	Explain the scientific background and rationale for the investigation being reported	Intro par 1-2	
Objectives	3	State specific objectives, including any prespecified hypotheses	Intro par 3	Last sentence
**Methods**				
Study design	4	Present key elements of study design early in the paper	Methods par 2	
Setting	5	Describe the setting, locations, and relevant dates, including periods of recruitment, exposure, follow-up, and data collection	Methods par 2	
Participants	6	(*a*) *Cohort study*—Give the eligibility criteria, and the sources and methods of selection of participants. Describe methods of follow-up<br>*Case-control study*—Give the eligibility criteria, and the sources and methods of case ascertainment and control selection. Give the rationale for the choice of cases and controls<br>*Cross-sectional study*—Give the eligibility criteria, and the sources and methods of selection of participants	Methods par 2	
		(*b*) *Cohort study*—For matched studies, give matching criteria and number of exposed and unexposed<br>*Case-control study*—For matched studies, give matching criteria and number of controls per case	N/A	
Variables	7	Clearly define all outcomes, exposures, predictors, potential confounders, and effect modifiers. Give diagnostic criteria, if applicable	Methods par 4	
Data sources/ measurement	8*	For each variable of interest, give sources of data and details of methods of assessment (measurement). Describe comparability of assessment methods if there is more than one group	Methods par 3	
Bias	9	Describe any efforts to address potential sources of bias	Methods par 5	(+ adjustment for confounders)
Study size	10	Explain how the study size was arrived at	Methods par 2	(+ suppl figure 1)

Continued on next page

Quantitative variables	11	Explain how quantitative variables were handled in the analyses. If applicable, describe which groupings were chosen and why	Methods par3 N/A
Statistical methods	12	(*a*) Describe all statistical methods, including those used to control for confounding	Methods par 5
		(*b*) Describe any methods used to examine subgroups and interactions	Methods par 5
		(*c*) Explain how missing data were addressed	Methods par 5
		(*d*) *Cohort study*—If applicable, explain how loss to follow-up was addressed *Case-control study*—If applicable, explain how matching of cases and controls was addressed *Cross-sectional study*—If applicable, describe analytical methods taking account of sampling strategy	No losses to follow-up
		(*e*) Describe any sensitivity analyses	Methods par 5
**Results**			
Participants	13*	(a) Report numbers of individuals at each stage of study—eg numbers potentially eligible, examined for eligibility, confirmed eligible, included in the study, completing follow-up, and analysed	Suppl figure 1
		(b) Give reasons for non-participation at each stage	Suppl figure 1
		(c) Consider use of a flow diagram	Suppl figure 1
Descriptive data	14*	(a) Give characteristics of study participants (eg demographic, clinical, social) and information on exposures and potential confounders	Table 1
		(b) Indicate number of participants with missing data for each variable of interest	Table 1 + description
		(c) *Cohort study*—Summarise follow-up time (eg, average and total amount)	Results par 3
Outcome data	15*	*Cohort study*—Report numbers of outcome events or summary measures over time	Results par 3
		Case-control study—Report numbers in each exposure category, or summary measures of exposure	*N/A*
		Cross-sectional study—Report numbers of outcome events or summary measures	*N/A*
Main results	16	(*a*) Give unadjusted estimates and, if applicable, confounder-adjusted estimates and their precision (eg, 95% confidence interval). Make clear which confounders were adjusted for and why they were included	Through results
		(*b*) Report category boundaries when continuous variables were categorized	N/A
		(*c*) If relevant, consider estimates of relative risk into absolute risk for a meaningful time period	N/A

Continued on next page

Other analyses	17	Report other analyses done—eg analyses of subgroups and interactions, and sensitivity analyses	Results par 3 (and methods par on statistics)
**Discussion**			
Key results	18	Summarise key results with reference to study objectives	Disc par 1
Limitations	19	Discuss limitations of the study, taking into account sources of potential bias or imprecision. Discuss both direction and magnitude of any potential bias	Disc par 6
Interpretation	20	Give a cautious overall interpretation of results considering objectives, limitations, multiplicity of analyses, results from similar studies, and other relevant evidence	Throughout discussion
Generalisability	21	Discuss the generalisability (external validity) of the study results	Discussion par 5-6
**Other information**			
Funding	22	Give the source of funding and the role of the funders for the present study and, if applicable, for the original study on which the present article is based	Disclosures

*Give information separately for cases and controls in case-control studies and, if applicable, for exposed and unexposed groups in cohort and cross-sectional studies.

Note: An Explanation and Elaboration article discusses each checklist item and gives methodological background and published examples of transparent reporting. The STROBE checklist is best used in conjunction with this article (freely available on the Web sites of PLoS Medicine at http://www.plosmedicine.org/, Annals of Internal Medicine at http://www.annals.org/, and Epidemiology at http://www.epidem.com/). Information on the STROBE Initiative is available at www.strobe-statement.org.

Supplemental Table 2 | Baseline characteristics of 628 KTR in tertiles of plasma SDMA

	Tertile 1 (N=209)	Tertile 2 (N=211)	Tertile 3 (N=208)	P-value
Plasma SDMA (µg/dL)	14 [13 to 15]	19 [18 to 21]	28 [25 to 33]	<0.001
Urea (mmol/L)	7.0 [5.9 to 8.7]	9.4 [7.6 to 11.3]	15.6 [11.6 to 19.2]	<0.001
**Clinical parameters**				
Female sex (n (%))	109 (52%)	85 (41%)	80 (38%)	0.011
Age at visit (years)	53 ± 13	53 ± 13	53 ±13	0.82
Pre-emptive transplantation (n (%))	37 (18%)	38 (18%)	24 (12%)	0.16
Time after transplantation (years)	5.4 [2.2 to 11.0]	4.9 [1.5 to 10.6]	5.8 [1.6 to 13.9]	0.28
History of rejection (n (%))	42 (20%)	50 (24%)	77 (37%)	<0.001
Primary renal disease (n (%))				0.14
Diabetic nephropathy	4 (2%)	11 (5%)	14 (7%)	
Glomerulonephritis	49 (23%)	57 (27%)	58 (28%)	
Interstitial nephritis	36 (17%)	22 (10%)	25 (12%)	
Cystic kidney disease	34 (16%)	53 (25%)	40 (19%)	
Renal vascular disease	14 (7%)	8 (4%)	11 (5%)	
Other congenital/hereditary disease	11 (5%)	11 (5%)	9 (4%)	
Other multisystem diseases	15 (7%)	15 (7%)	14 (7%)	
Other or unknown cause	46 (22%)	34 (16%)	37 (18%)	
Diabetes (n (%))	55 (26%)	50 (24%)	48 (23%)	0.85
History of CV disease (n (%))	70 (37%)	65 (35%)	84 (46%)	0.07
Body mass index (kg/m^2)	27.0 ± 4.9	26.8 ± 4.9	26.1 ± 4.6	0.26
Systolic blood pressure (mmHg)	135 ± 17	137 ± 17	137 ± 19	0.16
Current smoking (n (%))	29 (15%)	15 (7%)	29 (15%)	0.03
Donor age (years)	40 [27 to 52]	49 [34 to 56]	49 [38 to 56]	<0.001
Living donor (n (%))	82 (39%)	90 (43%)	49 (24%)	<0.001
**Laboratory measurements**				
Leukocytes (x 10^9/L)	8.3 ± 2.4	8 ± 2.4	8.2 ± 3.1	0.34
Hemoglobin (mmol/L)	8.7 ± 0.9	8.2 ± 1	7.6 ± 1	<0.001
Sodium (mmol/L)	141.3 ± 2.3	140.8 ± 2.6	140.3 ± 3.1	<0.001
Potassium (mmol/L)	3.8 ± 0.4	4 ± 0.4	4.2 ± 0.5	<0.001
eGFR (mL/min/1,73m2)	71.5 ± 16.0	51.8 ± 12.9	33.6 ± 11.2	<0.001
Creatinine (µmol/L)	96 [82 to 110]	122 [107 to 147]	179 [145 to 222]	<0.001
hs-CRP (mg/L)	1.5 [0.8 to 4.9]	1.5 [0.7 to 3.9]	1.6 [0.7 to 4.6]	0.36
24h urinary protein excretion (g/24h)	0.1 [0 to 0.3]	0.2 [0 to 0.4]	0.3 [0.1 to 0.6]	<0.001
**Medication use** (n (%))				
Diuretics	54 (26%)	78 (37%)	119 (58%)	<0.001
RAAS-blocker	89 (43%)	102 (48%)	119 (57%)	0.005
Calcium-antagonist	57 (27%)	42 (20%)	55 (26%)	0.27
Beta blocker	131 (63%)	123 (59%)	144 (69%)	0.07
Prednisolone	207 (99%)	209 (99%)	205 (99%)	0.86
Calcineurin inhibitor	91 (44%)	130 (62%)	150 (72%)	<0.001
Proliferation inhibitor	183 (88%)	175 (83%)	163 (78%)	0.04
mTOR inhibitor	10 (5%)	6 (3%)	4 (2%)	0.20

Normally distributed data are presented as means ± standard deviation, skewed data as median [interquartile range], and categorical data as number (valid percentage). P-values represent significance of differences between tertiles as assessed with Analysis of Variance, Kruskal-Wallis or Chi-squared tests, depending on distribution. CV, cardiovascular; eGFR, estimated glomerular filtration rate as calculated using CKD-EPI formula; hs-CRP, high-sensitivity C-reactive protein; RAAS, renin-angiotensin-aldosterone system; SDMA, symmetric dimethyl arginine.

Supplemental Table 3 | Cox regression analysis of the association of plasma SDMA and urea with graft failure-censored mortality

	Urea		SDMA	
**Model**	**HR per doubling (95% CI)**	**P-value**	**HR per doubling (95% CI)**	**P-value**
**Crude**	2.21 (1.71 to 2.86)	<0.001	2.83 (1.91 (4.20)	<0.001
**Model 1**	2.19 (1.68 to 2.86)	<0.001	2.90 (1.95 to 4.31)	<0.001
**Model 2**	2.41 (1.50 to 3.87)	<0.001	4.82 (2.42 to 9.59)	<0.001
**Model 3**	2.19 (1.34 to 3.58)	0.002	4.31 (2.19 to 8.46)	<0.001
**Model 4**	2.11 (1.28 to 3.50)	0.004	4.03 (2.06 to 7.91)	<0.001
**Model 5**	2.11 (1.26 to 3.51)	0.005	4.03 (2.04 to 7.93)	<0.001
**Model 6**	1.75 (1.03 to 2.99)	0.04	3.30 (1.66 to 6.56)	<0.001
**Model 7**	1.34 (0.78 to 2.32)	0.32	2.98 (1.46 to 6.09)	0.003

During follow-up, 111 patients (18%) experienced graft failure-censored death during a median follow-up of 5.4 [4.5 to 6.0] years. Model 1, adjustment for age, sex and time after transplantation. Model 2, adjustment for variable in model 1 + creatinine-based eGFR + living vs. deceased donor status + donor age + pre-emptive transplantation. Model 3, adjustment for model 2 +rejection + 24h urinary protein excretion. Model 4, adjustment for model 3 + diabetes + history of cardiovascular disease. Model 5, adjustment for model 4 + diuretic use + calcineurin inhibitor use. Model 6, adjustment for model 5 + albumin + hemoglobin + high-sensitivity C-reactive protein + N-terminal pro-brain natriuretic peptide. Model 7, adjustment for model 6 + urea (in case of SDMA) or SDMA (in case of urea). Abbreviations: CI, confidence interval; HR, hazard ratio; SDMA, symmetric dimethyl arginine

Supplemental Table 4 | Cox regression analysis of the association of plasma SDMA with cause-specific mortality

Cause of death	Cardiovascular		Malignant		Infectious		Miscellaneous	
	**HR per doubling (95% CI)**	**P**	**HR per doubling (95% CI)**	**P**	**HR per doubling (95% CI)**	**P**	**HR per doubling (95% CI)**	**P**
**SDMA**								
Crude	2.70 (1.58 to 4.61)	<0.001	1.89 (0.81 to 4.41)	0.13	3.00 (1.64 to 5.49)	<0.001	3.07 (1.35 to 7.00)	0.01
Model 1	3.00 (1.71 to 5.24)	<0.001	2.24 (0.93 to 5.42)	0.07	3.45 (1.85 to 6.43)	<0.001	3.89 (1.59 to 9.11)	0.005
**Urea**								
Crude	2.66 (1.73 to 4.08)	<0.001	1.37 (0.69 to 2.71)	0.35	2.12 (1.31 to 3.43)	0.003	2.52 (1.30 to 4.89)	0.008
Model 1	2.68 (1.71 to 4.20)	<0.001	1.33 (0.65 to 2.71)	0.41	2.15 (1.31 to 3.51)	0.003	2.61 (1.28 to 5.33)	0.01

During follow-up, 51 patients (8%) experienced death from cardiovascular causes, 41 (6%) from infectious causes, 23 (4%) from malignancy, and 23 (4%) from miscellaneous causes during the median follow-up of 5.5 [4.7 to 6.1] years. **Model 1**, adjustment for age and sex, no further adjustments because of limited numbers of events. Abbreviations: CI, confidence interval; HR, hazard ratio; SDMA, symmetric dimethyl arginine.

Supplemental Figure 1 | Diagram visualizing the flow of participants through the study

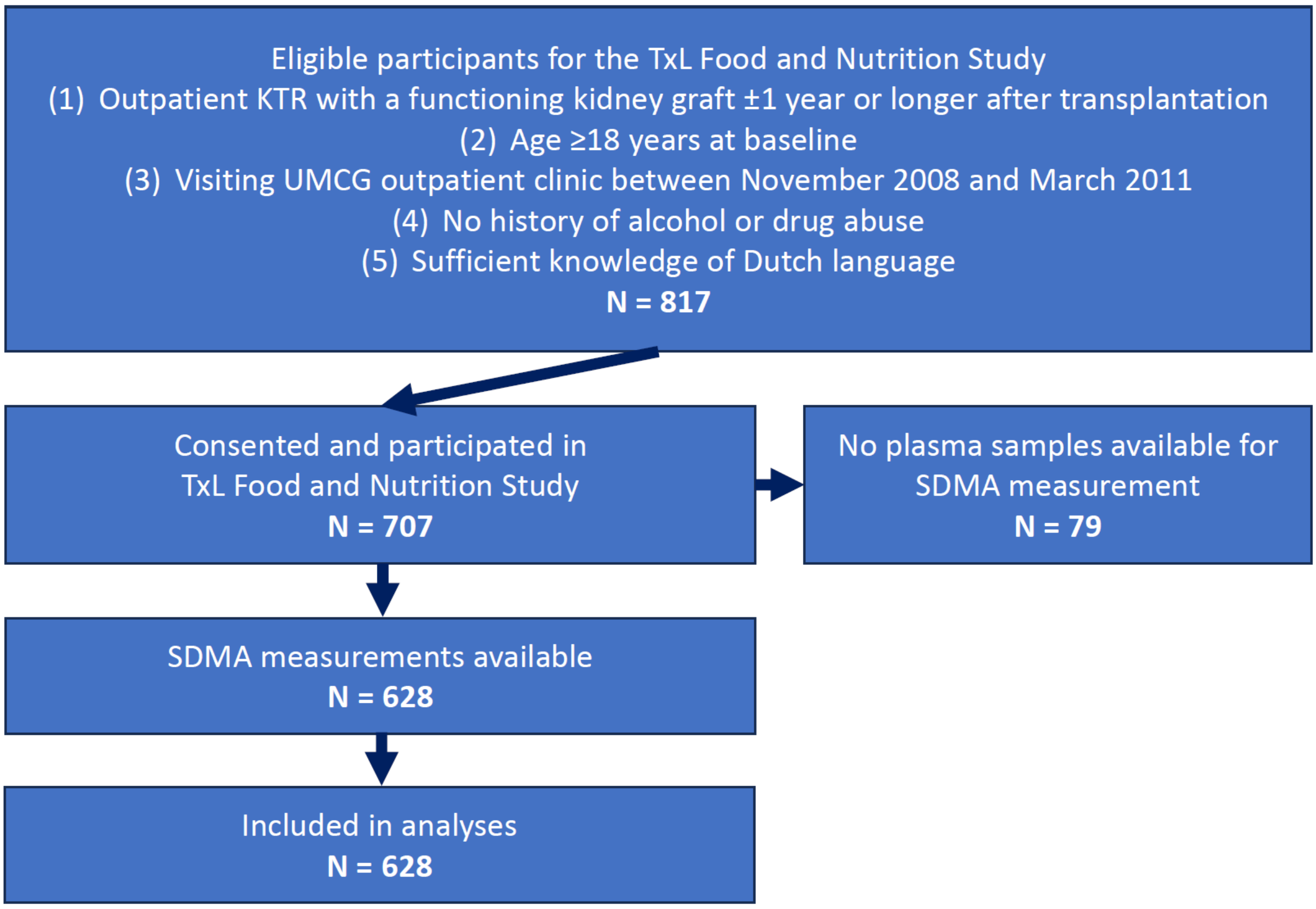

Supplemental Figure 2 | Kaplan Meier curve visualizing differences in survival across SDMA and urea tertiles

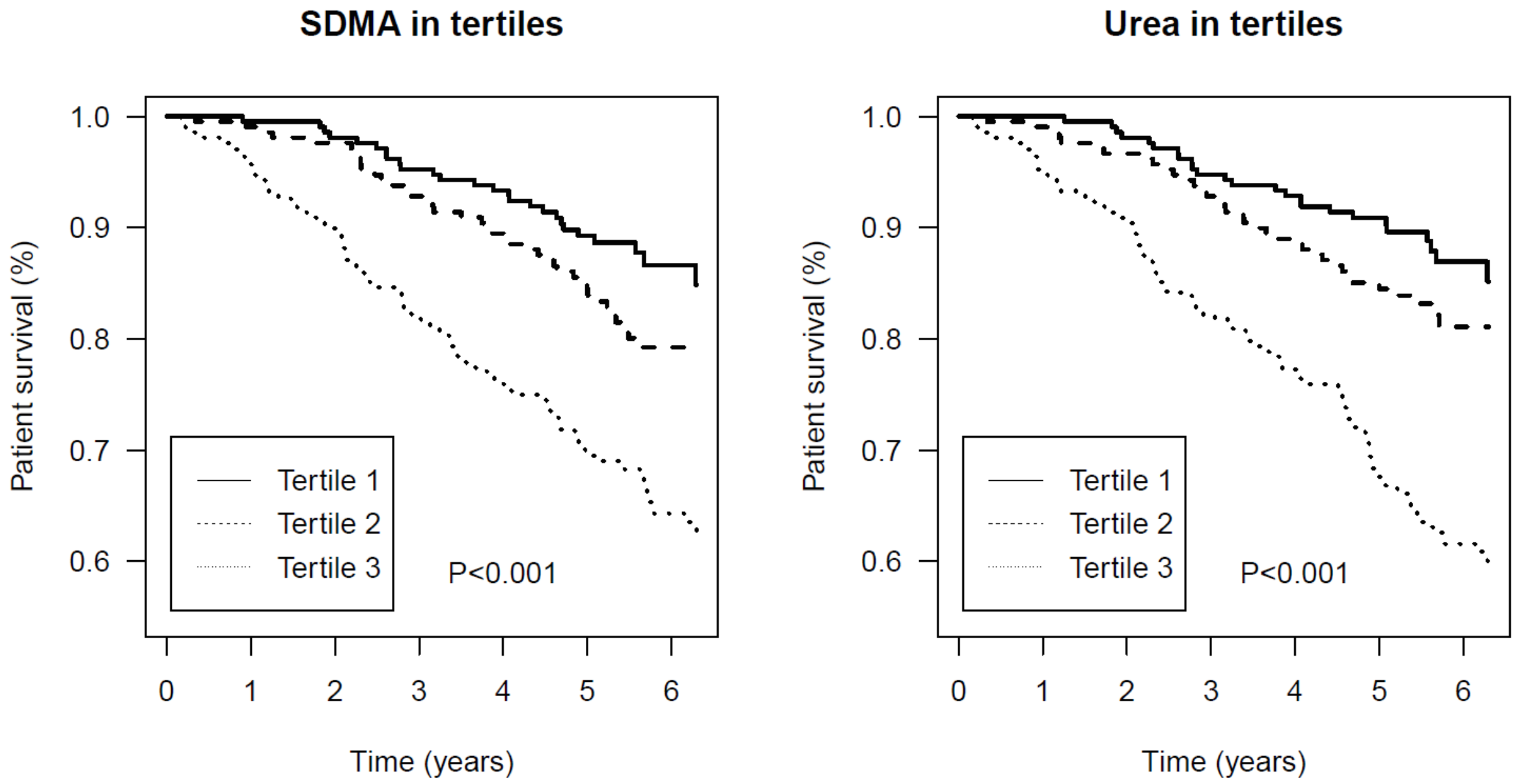

SDMA, symmetric dimethyl arginine. P-values indicate statistical significance of between-group differences as calculated using log-rank tests
